# CO_2_ hydrogenation to high-value products via heterogeneous catalysis

**DOI:** 10.1038/s41467-019-13638-9

**Published:** 2019-12-13

**Authors:** Run-Ping Ye, Jie Ding, Weibo Gong, Morris D. Argyle, Qin Zhong, Yujun Wang, Christopher K. Russell, Zhenghe Xu, Armistead G. Russell, Qiaohong Li, Maohong Fan, Yuan-Gen Yao

**Affiliations:** 1 Departments of Chemical and Petroleum Engineering, University of Wyoming, Laramie, WY 82071 USA; 2 Key Laboratory of Coal to Ethylene Glycol and Its Related Technology, Fujian Institute of Research on the Structure of Matter, Chinese Academy of Sciences, Fuzhou, Fujian, 350002 China; 3 University of Chinese Academy of Sciences, Beijing, 100049 China; 4 School of Chemical Engineering, Nanjing University of Science and Technology, Nanjing, Jiangsu 210094 P.R. China; 5 Department of Chemical Engineering, Brigham Young University, 330 EB, Provo, UT 84602 USA; 6 State Key Laboratory of Chemical Engineering, Department of Chemical Engineering, Tsinghua University, Beijing, 100084 P.R. China; 7 Departments of Civil and Environmental Engineering, Stanford University, Stanford, 94305 CA USA; 8 Department of Materials Science and Engineering, Southern University of Science and Technology, Shenzhen, 518055 China; 9 School of Civil and Environmental Engineering, Georgia Institute of Technology, Mason Building, 790 Atlantic Drive, Atlanta, GA 30332 USA; 10 School of Energy Resources, University of Wyoming, Laramie, WY 82071 USA

**Keywords:** Catalytic mechanisms, Heterogeneous catalysis, Chemical engineering

## Abstract

Recently, carbon dioxide capture and conversion, along with hydrogen from renewable resources, provide an alternative approach to synthesis of useful fuels and chemicals. People are increasingly interested in developing innovative carbon dioxide hydrogenation catalysts, and the pace of progress in this area is accelerating. Accordingly, this perspective presents current state of the art and outlook in synthesis of light olefins, dimethyl ether, liquid fuels, and alcohols through two leading hydrogenation mechanisms: methanol reaction and Fischer-Tropsch based carbon dioxide hydrogenation. The future research directions for developing new heterogeneous catalysts with transformational technologies, including 3D printing and artificial intelligence, are provided.

## Abstract (web summary)

Carbon dioxide (CO_2_) capture and conversion provide an alternative approach to synthesis of useful fuels and chemicals. Here, Ye et al. give a comprehensive perspective on the current state of the art and outlook of CO_2_ catalytic hydrogenation to the synthesis of light olefins, dimethyl ether, liquid fuels, and alcohols.

## Introduction

Capturing carbon dioxide (CO_2_) has emerged as an important method of mitigating its impact on environment^1–5^. However, this introduces the challenge of utilizing a large volume of captured CO_2_, which previously has not had any industrially viable uses at such a large scale. Realizing that, historically, fossil resources were produced via natural carbon-hydrogenation during photosynthesis, synthetic CO_2_ hydrogenation is likely the best way to regenerate combusted hydrocarbons.

While CO_2_ hydrogenation is challenging due to the thermal stability of CO_2_, making reaction conversions low, considerable progress has been made towards converting CO_2_ to single carbon (C_1_) products (e.g., formic acid, carbon monoxide (CO), methane, and methanol) through direct hydrogen reduction or hydrothermal-chemical reduction in water^6–11^. Thermocatalytic hydrogenation of CO_2_ to methane can be achieved easily at atmospheric pressure and high gas hourly space velocity (GHSV), and has been shown to achieve CO_2_ conversion and CH_4_ selectivity close to theoretical equilibrium values^12^. Hydrogenation to CO can be performed via the reverse water gas shift (RWGS) reaction. CO_2_-to-methanol (CTM) (CH_3_OH and MeOH) has already been industrialized in Reykjavik, Iceland using heterogeneous catalysis and geothermal energy^13^.

In addition, catalysis for CO_2_ hydrogenation to C_1_ products has been widely studied with considerable progress^14^. Recently, significant progress has been achieved in heterogeneous catalytic hydrogenation of CO_2_ to various high-value and easily marketable fuels and chemicals containing two or more carbons (C_2+_ species), including dimethyl ether (DME)^15^, olefins^16^, liquid fuels^17^, and higher alcohols^18^. Compared to C_1_ products, C_2+_ product synthesis is more challenging due to the extreme inertness of CO_2_ and the high C–C coupling barrier, as well as many competing reactions leading to the formation of C_1_ products. However, C_2+_ hydrocarbons and oxygenates possess higher economic values and energy densities than C_1_ compounds. Therefore, this perspective mainly focuses on heterogeneous catalysis for CO_2_ hydrogenation to high-value C_2+_ products.

Many reviews of heterogeneous catalytic hydrogenation of CO_2_ have been published, organized according to using several approaches, including thermal, electrochemical, and photochemical hydrogenation^19^; homogeneous and heterogeneous catalysts^20^; and by their respective product distributions or catalysts employed^14,21–24^. Some are limited to C_1_ products (e.g., methane or methanol)^14,25^. However, perspectives on heterogeneous catalytic CO_2_ hydrogenation to C_2+_ products are needed to guide scientists working in this area. Thus, this work is designed to help fill this gap and is organized to emphasize reaction mechanisms for producing C_2+_ materials.

The hydrogenation of CO_2_ to C_2+_ products mainly occurs via a methanol-mediated route or a CO_2_ modified Fischer–Tropsch mechanism^17,26^. There are a variety of questions that need to be answered for each of these reaction mechanisms: what kinds of catalysts are beneficial for each route? How do the catalysts regulate product selectivity? What can be done to further enhance catalytic performance? What is the central challenge with catalysts for CO_2_ hydrogenation to C_2+_ products? To answer these questions, the catalysts for CO_2_ hydrogenation to C_2+_ species will be discussed based on the methanol-mediated route and the CO_2_ modified Fischer–Tropsch route. Some experimental guidelines are provided to improve CO_2_ conversion and to reduce C_1_ byproducts. In addition, an outlook of transformational technologies for developing new catalysts is given in this work. Artificial intelligence (AI) is expected to guide the design and discovery of catalysis^27,28^, while 3D printing technologies are anticipated to be used to manufacture them on a large scale^29^.

## Methanol reaction based CO_2_ hydrogenation

**Fig. 1:**
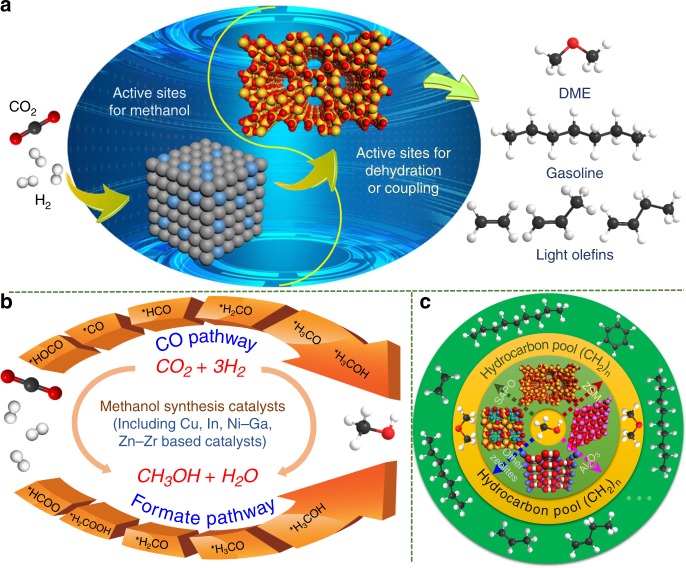
CO_2_ hydrogenation to fuels and chemicals via the methanol reaction mechanism. **a** Schematic for the reaction mechanism of direct CO_2_ hydrogenation to C_2+_ products over bifunctional catalysts. **b** Two possible reaction pathways for methanol synthesis. **c** Schematic for methanol conversion into hydrocarbons inside zeolites via the hydrocarbon-pool mechanism.

Methanol (CH_3_OH) reaction based CO_2_ hydrogenation can be realized by coupling two sequential reactions over a bifunctional catalyst. First, CO_2_ and H_2_ are converted to CH_3_OH over a partially reduced oxide surface (e.g., Cu, In, and Zn) or noble metals via a CO or formate pathway. Then, methanol is dehydrated or coupled over zeolites or alumina. Accordingly, bifunctional or hybrid catalysts are composed of a CH_3_OH synthesis catalyst and a CH_3_OH dehydration/coupling catalyst, which can convert CO_2_ into high-value C_2+_ compounds, including DME, hydrocarbons like gasoline, and light olefins. An efficient catalyst for these high-value C_2+_ products should be active for both CH_3_OH synthesis and dehydration/coupling under the same conditions (Fig. 1a, R1–R4).

R1 $${\mathrm{CO}}_2 + 3{\mathrm{H}}_2 \to {\mathrm{CH}}_3{\mathrm{OH}} + {\mathrm{H}}_2{\mathrm{O}}$$

R2 $$2{\mathrm{CH}}_3{\mathrm{OH}} \to {\mathrm{CH}}_3{\mathrm{OCH}}_3 + {\mathrm{H}}_2{\mathrm{O}}$$

R3 $${n}{\mathrm{CH}}_3{\mathrm{OH}} + {\mathrm{H}}_2 \to {\mathrm{CH}}_3\left( {{\mathrm{CH}}_2} \right)_{n - 2}{\mathrm{CH}}_3 + {{n}\mathrm{H}}_2{\mathrm{O}}$$

R4 $${n}{\mathrm{CH}}_3{\mathrm{OH}} \to {\mathrm{CH}}_2 = {\mathrm{CH}}\left( {{\mathrm{CH}}_2} \right)_{n - 3}{\mathrm{CH}}_3 + {{n}\mathrm{H}}_2{\mathrm{O}}$$

In CO_2_ hydrogenation to C_2+_ compounds, the reactions of CO_2_ to CH_3_OH and CH_3_OH to C_2+_ compounds take place at 200–300 °C and 400 °C, respectively, over bifunctional catalysts. Therefore, investigation of the reaction conditions, catalyst properties, and catalytic performance for CO_2_ to CH_3_OH and CH_3_OH to C_2+_ products is necessary.

### Methanol

As mentioned earlier, one of the possible targets of CO_2_ hydrogenation is to convert CO_2_ to CH_3_OH. Significant progress has been made in this area recently, especially in developing copper (Cu)- and indium (In)-based catalysts^30,31^. Recent publications have dealt with Cu–ZnO composites, whose CH_3_OH selectivity ranges from 30 to 70% at CO_2_ conversions less than 30% under typical reaction conditions (temperature: 220–300 °C, pressure <5 MPa, H_2_/CO_2_ = 3)^32,33^. By increasing the pressure to 36 MPa and H_2_/CO_2_ molar ratio to 10, the single pass CO_2_ conversion can be increased to 95.3% with 98.2% methanol selectivity over a Cu–ZnO–Al_2_O_3_ catalyst^34^. When Cu nanoparticles (NPs) are encapsulated in metal organic frameworks (MOFs) and strongly interact with their secondary structural units, the resultant metal NPs@MOFs (e.g., Cu ⊂ UiO-66^35^ and CuZn@UiO-bpy^36^) show enhanced activity and 100% CH_3_OH selectivity, while preventing the agglomeration of the Cu NPs. In addition, ZrO_2_ is a noted promoter or support in Cu-based catalysts for CTM hydrogenation^37,38^.

Indium-based materials have shown promise as alternatives for CO_2_ conversion to methanol. Pure In_2_O_3_ can convert 7.1% of CO_2_ with 39.7% selectivity to CH_3_OH at 330 °C and 5 MPa^39^. A Pd/In_2_O_3_ catalyst with many interfacial sites and oxygen vacancies enhances CO_2_ adsorption, achieving CO_2_ conversions of above 20% with a methanol space time yield (STY) of 0.89 g_MeOH_ g_cat_^−1^ h^−1^ at 300 °C and 5 MPa^31^. In_2_O_3_/ZrO_2_ catalysts significantly boost the methanol selectivity to 99.8%, with a CO_2_ conversion of 5.2% and long-term stability of 1000 h under industrially relevant conditions^40^. Besides the Cu and In-based catalysts, great progress has also been achieved with ZnO–ZrO_2_ solid solution catalysts, as well as Pd/Pt-based catalysts^41–43^. The ZnO–ZrO_2_ solid solution catalysts have achieved CH_3_OH selectivities of 86–91% under a GHSV of 24,000 mL g^−1^ h^−1^ with high thermal stability after being tested for more than 500 h; it also shows a strong resistance to sulfur-containing molecules^31^. In 2014, Norskov’s group reported a Ni–Ga bimetallic catalyst for CO_2_ hydrogenation to CH_3_OH at 0.1 MPa, whose STY reached 0.64 g_MeOH_ g_cat_^−1^ h^−1^ at 210 °C^44^. Subsequently, a series of noble metal based catalysts, such as Ga–Pd^45^, Au–CeO_X_/TiO_2_^46^, and Pt–MoO_X_/Co-TiO_2_^47^, were developed to convert CO_2_ to CH_3_OH at low pressure or low temperature. Similarly, Fan’s group achieved low-pressure CH_3_OH synthesis via another series of multiple-metal catalysts that included In_2_O_3_, like Ni–In–Al/SiO_2_ and La–Ni–In–Al/SiO_2_, starting with a phyllosilicate precursor^3,48^, and achieving CH_3_OH STYs of above 0.011 g_MeOH_ g_cat_^−1^ h^−1^ at 255 °C and 0.1 MPa.

### Dimethyl ether (DME)

There has been rapid development in DME synthesis from CO_2_ hydrogenation using CH_3_OH synthesis catalysts hybridized with CH_3_OH coupling catalysts^15^. The effect of promoters, supports, and synthesis conditions have been explored^42,43,49^. For example, the acidic sites on γ-alumina surfaces and the CuAl_2_O_4_ spinel phase can be regulated by promoters like gallium or zinc oxides, resulting in higher stability for Cu NPs during CO_2_-to-DME^49^. To date, CO_2_ conversion and DME selectivity mostly vary between 35–80% and 5–50%, respectively^15^, but CO_2_ conversion can reach up to 97% at 280 °C over a Cu–Zn–Al/HZSM-5 catalyst by drastically increasing the reaction pressure (to 36 MPa)^34^. In addition, interesting results have been reported on core–shell structured hybrid catalysts with the MeOH synthesis catalysts at the core and the MeOH dehydration catalysts forming the shell^50,51^. Compared with traditional hybrid catalysts prepared by physically mixing the components, these novel core–shell catalysts have received much attention in the literature due to their unique structures and ability to valorize CO_2_ by improving conversion and DME selectivity.

### Light olefins, aromatics, and gasoline

MeOH can not only be dehydrated to DME, but also serve as an intermediate to synthesize hydrocarbon chains, (CH_2_)_*n*_, and final products, such as olefins, aromatics, and gasoline. As mentioned above, defective indium oxide (In_2_O_3_) with oxygen vacancies is shown to be effective for CO_2_ hydrogenation to CH_3_OH, while In_2_O_3_ mixed with SAPO-34 is attractive for efficient CO_2_ conversion to CH_3_OH and subsequent selective C–C coupling of CH_3_OH to form light olefins^52^. The addition of Zr into In_2_O_3_ is helpful for creating more oxygen vacancies, enhancing CO_2_ chemisorption and stabilizing both surface intermediates and active In NPs^53,54^. Similarly, a high yield of light olefins can also be achieved by composite catalysts, such as ZnZrO^16^, ZnGaO^55^, and CuZnZr^56^ mixed with SAPO-34. As shown in Table 1 (Entries 1–5, 9–13), the selectivity of light olefins, represented by the C_2_–C_4_^=^ column in the table, can be as high as 90%, while the CH_4_ selectivity is less than 5% of the hydrocarbon products with 15–30% CO_2_ conversions over most bifunctional catalysts tested, which deviates from the Anderson–Schulz–Flory (ASF) distribution.^52,54^ This result is a significant breakthrough in the synthesis of light olefins. When the zeolite is changed from SAPO-34 to HZSM-5, more C_5+_ compounds are produced than light olefins. A 78.6% selectivity of gasoline-range hydrocarbons, with only 1% CH_4_ selectivity, was obtained from the tandem In_2_O_3_/HZSM-5 catalyst^26^. When In_2_O_3_ is replaced by ZnAlO_*x*_ or ZnZrO, CH_3_OH is synthesized on the metal oxide surface and then converted to olefins and aromatics inside the HZSM-5 pores with an aromatic selectivity of 73%, which is attributed to a shielding of the external Brønsted acid sites of HZSM-5 by ZnAlO_*x*_^57^. Therefore, the type of product is affected by both the character of the metal oxides and the geometries of the zeolites that determine the confinement of the hydrocarbons. Furthermore, as mentioned previously, catalysts with product yields exceeding the ASF distribution limit were recently reported that follow the methanol reaction mechanism-based CO/CO_2_ hydrogenation. However, fundamental understanding of this ASF distribution deviation is still lacking. Jiao et al.^58^ observed that ketene (CH_2_CO) can be formed from the reaction between surface CO and CH_2_ species, blocking the surface polymerization, and thus breaking the ASF distribution. Another important reason suggested for the reported ASF distribution deviation is the use of bifunctional catalysts with two types of active sites.

**Table 1:** Representative catalysts and their performance for hydrogenation of CO_2_ to C_2+_ species.

Entry	Catalysts	CO_2_ con./%	CO select./%	CH select./%	Hydrocarbon distribution/%^a^				GHSV/ml g^−1^ h^−1^	Temp./°C	P/MPa	Ref.
					CH_4_	C_2_–C_4_^0^	C_2_–C_4_^=^	C_5+_				
*C*_*2+*_ *hydrocarbons based on methanol reaction mechanism*												
1	In_2_O_3_/ZrO_2_+SAPO-34	19.0	87.0	13.0	4.0	12.0	84.0	–	3000	400	1.5	^53^
2	In_2_O_3_/SAPO-34	15.3	68.3	31.7	2.7	13.7	81.9	1.7	9000	380	3.0	^52^
3	In_2_O_3_–ZrO_2_/SAPO-34	26.2	63.9	36.1	2.0	21.5	74.5	2.0	9000	380	3.0	^52^
4	In–Zr/SAPO-34	29.0	78.2	–	4.1	9.2	83.9	2.8	15,750	400	3.0	^54^
5	ZnZrO/SAPO-34	12.6	47.0	–	3.0	14.0	80.0	3.0	3600	380	2.0	^16^
6	(CuO–ZnO)–Kaolin/SAPO-34	50.4	7.5	–	13.6	15.8	70.6	0.0	1800	400	3.0	^145^
7	Zn–Ga–O/SAPO-34	13.0	46.0	–	1.0	11.0	86.0	2.0	5400	370	3.0	^55^
8	CuZnZr@Zn–SAPO-34	19.6	58.6	41.4	14.6	20.2	60.5	4.8	3000	400	2.0	^56^
9	In_2_O_3_/HZSM-5	13.1	44.8	–	1.0	–	–	78.6	9000	340	3.0	^26^
10	Cr_2_O_3_/HZSM-5	33.6	41.2	–	3.0	15.7	3.1	78.2	1200	350	3.0	^146^
11	Fe_2_O_3_/HZSM-5	7.1	73.5	–	2.0	–	–	70.5	9000	340	3.0	^26^
12	ZnAlO_*x*_ and HZSM-5	9.1	57.4	42.6	0.5	6.7	10.7	80.3	2000	320	3.0	^57^
13	ZnZrO/HZSM-5	14.1	43.7	57.3	0.3	14.5	4.9	80.3	1200	320	4.0	^71^
*C*_*2+*_ *hydrocarbons based on CO*_*2*_ *modified FTS mechanism*												
14	FeZnK–NC	34.6	21.2	78.8	24.2	7.1	40.6	28.1	7200	320	3.0	^120^
15	Fe–2K	~30.0	22.0	74.0	31.1	14.9	32.4	21.6	–	320	2.0	^82^
16	10Fe0.8K0.53Co	54.6	2.0	98.0	19.3	7.8	24.9	48.0	560	300	2.5	^91^
17	N–K–600-0	43.1	26.1	73.9	35.5	6.8	36.9	20.8	3600	400	3.0	^121^
18	1Fe–1Zn–K	51.0	6.0	85.1	34.9	7.8	53.6	3.7	1000	320	0.5	^147^
19	35Fe–7Zr–1Ce–K	57.3	3.1	96.3	20.6	7.9	55.6	15.9	1000	320	2.0	^76^
20	Fe–Co/K–Al_2_O_3_	41.4	14.8	85.2	21.7	6.3	45.0	27.0	9000	320	3.0	^77^
21	C–Fe–Zn/K	54.8	4.6	94.4	23.1	8.5	57.4	11.0	1000	320	2.0	^78^
22	Na–Fe_3_O_4_/HZSM-5	22.0	20.1	–	4.0	–	–	79.4	4000	320	3.0	^17^
23	ZnFeO_*x*_–4.25Na/S–HZSM-5	36.2	11.0	89.0	8.2	13.3	3.2	75.4	4000	320	3.0	^93^
24	Fe–Cu–K–La/TiO_2_	23.1	33.0	67.0	19.4	–	–	67.2	3600	300	1.1	^115^
25	Na–ZnFe_2_O_4_	34.0	11.7	–	9.7	–	–	58.5	1800	340	1.0	^116^
26	K–Fe	43.9	10.1	89.9	12.2	–	–	56.6	750	300	1.5	^148^
27	92.6Fe7.4 K	41.7	6.0	94.0	10.9	23.0	6.5	59.6	560	300	2.5	^122^
28	10Fe4.8 K	35.2	9.0	91.0	8.1	4.3	16.4	71.2	560	300	2.5	^91^
29	CuFeO_2_−24	16.7	31.4	–	2.4	–	–	64.9	1800	300	1.0	^123^
30	Na–CoCu/TiO_2_	18.4	30.2	–	26.1	–	–	42.1	3000	250	5.0	^96^
31	Co/MIL-53(Al)	25.3	6.6	18.7	35.2	–	–	35.0	800	260	3.0	^94^

Entry	Catalysts	CO_2_ con./%	CO select./%	HC select./%	MeOH select./%	C_2+_OH select./%	GHSV/ml g^−1^ h^−1^	Temp./°C	P/MPa	Ref.
*C*_*2+*_ *alcohols from CO*_*2*_ *hydrogenation*										
32	CuZnFe_0.5_K_0.15_	42.3	6.9	56.4	4.7	32.0, C_2+_OH	5000	300	6.0	^99^
33	Mo_1_Co_1_K_0.8_ sulfide	28.8	–	–	70.9	10.9, C_2+_OH	3000	320	5.0	^149^
34	1 wt%Pt/Co_3_O_4_	–	–	–	–	82.5, C_2+_OH	–	200	8.0	^98^
35	PdCu NPs/P25	–	–	–	–	92.0, EtOH	–	200	3.2	^150^
36	RhFeLi/TiO_2_	15.7	12.5	53.9	2.2	31.3, EtOH	6000	250	3.0	^18^

^a^The hydrocarbon distribution was calculated without CO

### Reaction mechanisms

For most catalysts, the rate-determining step for methanol synthesis is CO_2_ activation (R1)^59,60^, which includes chemisorption of CO_2_ and electron transfer from the catalyst to CO_2_^11,61^. Density functional theory (DFT) calculations have shown that different catalysts activate CO_2_ through electron transfer between different orbitals^62^. For example, in PtCo-based catalysts, carbon in the CO_2_ tends to bind to the Pt sites with an η^1^-C_Pt_ bonding mode, while the O in the CO_2_ prefer to combine with the reduced M^δ+^ cations in metal oxides with η^1^-OM^δ+^ configuration^63^. In addition, oxygen vacancies on metal oxide surfaces and Lewis acid sites have also been shown to enhance CO_2_ activation^37,55^, stabilize intermediates^64^, and reduce sintering^52^. DFT calculations reveal that an oxygen-defective surface could be created through direct thermal desorption or exposure to a reducing agent^54^. After activation, CO_2_ proceeds to form methanol, most likely via a formate intermediated pathway.

The use of DFT and ab initio molecular dynamics sampling techniques on Cu–ZnO^32^, Cu–Pd^42,65^, In^66^, and Ga^48,67^ catalysts suggest that methanol synthesis occurs via a formate intermediate (See Fig. 1b). However, while calculations for Cu–ZnO interfacial sites suggest a formate intermediate^32^, calculations for the near surface regions suggest a CO intermediated pathway^68^. In addition, the calculations indicate that H_2_O produced in situ from both RWGS and CH_3_OH formation on Pd incorporated Cu catalysts accelerates CO_2_ conversion to methanol by reducing the kinetic barriers by 0.2–0.7 eV for O–H bond formation steps^42,65^. On In-based catalysts, defective In_2_O_3_ surfaces have different CO_2_ activation barriers^66^. The synthesized methanol can then be transformed into high-value C_2+_ compounds, including DME, lower hydrocarbons, and gasoline via the hydrocarbon-pool mechanism in which an organic center is trapped in the zeolite pores and acts as a co-catalyst, as depicted in Fig. 1c^26,69,70^. The DFT results indicate that the rate-determining step is generally CO_2_ activation during conversion to methanol over the hybrid catalysts, whose ΔG^0^ at 320 °C reaches 47 kJ/mol, which is much higher than that of the subsequent dehydration reaction (e.g., ΔG^0^ = −87 kJ/mol for methanol to xylene at 320 °C)^71,72^; thus, an efficient CH_3_OH synthesis catalyst is beneficial for the subsequent formation of C_2+_ compounds.

As indicated above, the bifunctional catalysts consist of CH_3_OH synthesis and dehydration catalysts. However, is the bifunctional catalyst mechanism also the simple sum of the two individual reactions? Li et al.^16^ estimated the CH_3_OH selectivity based on the hydrocarbon selectivity for a hybrid catalyst and found that it was much higher than that for ZnZrO alone. This result clearly indicates that the products from the bifunctional catalysts are not a simple sum of the two individual reactions, and these two catalysts have a large synergistic effect. Thus, further study on the direct CO_2_ hydrogenation to C_2+_ products over bifunctional catalysts is still desirable.

## Fischer–Tropsch synthesis (FTS)-based CO_2_ hydrogenation

**Fig. 2:**
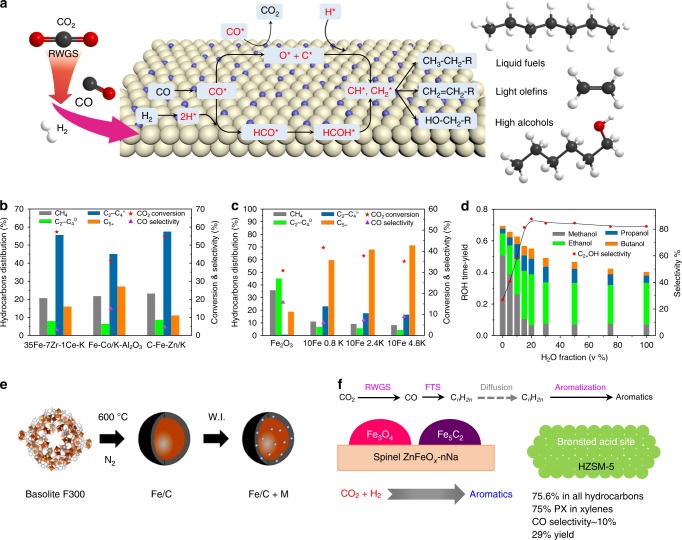
CO_2_ hydrogenation to C_2+_ products via the FTS-based mechanism. **a** Scheme of CO_2_ modified FTS-based catalytic mechanism. **b**–**d** CO_2_ hydrogenation via the FTS mechanism for production of light olefins, liquid fuels, and higher alcohols^76–78,91,98^. **e** Synthesis strategy for an Fe-based catalyst. (Reprinted with permission from Ramirez et al.^83^. Copyright (2018) American Chemical Society). **f** Selective production of aromatics from the CO_2_ hydrogenation process over a ZnFeO_*x*_–*n*Na/HZSM-5 catalyst. (Reprinted with permission from Cuiet al.^93^. Copyright (2019) American Chemical Society).

Heterogeneous FTS-based CO_2_ hydrogenation can be realized in either one or two reactors, although the single reactor process dominates. The direct one-reactor conversion system has drawn much attention due to its ease of operation and thus lower CO_2_ conversion cost. This process integrates the reduction of CO_2_ to CO via the RWGS reaction and hydrogenation of CO to hydrocarbons via FTS. An efficient catalyst for generating C_2+_ products, which generally refer to light olefins, liquid fuels, and higher alcohols, should be active for both RWGS and FTS under the same conditions (Fig. 2a, R5–R7). The product distribution can be wide, depending on the structure and composition of the catalysts. Iron (Fe), cobalt (Co), and ruthenium (Ru) based supported catalysts, with appropriate promoters, are predominantly used in this area.

R5 $${\mathrm{nCO}}_2 + \left( {3{\mathrm{n}} + 1} \right){\mathrm{H}}_2 \to {\mathrm{C}}_{\mathrm{n}}{\mathrm{H}}_{{\mathrm{2n + 2}}} + {\mathrm{2nH}}_{\mathrm{2}}{\mathrm{O}}\quad \left( {{\mathrm{CO}}_2\,{\mathrm{Hydrogenation}}} \right)$$

$$\Delta _{\mathrm{R}}{\mathrm{H}}_{{\mathrm{573K}}} = - 128\,{\mathrm{kJ/mol}}$$

R6 $${\mathrm{CO}}_{\mathrm{2}} + {\mathrm{H}}_2 \to {\mathrm{CO}} + {\mathrm{H}}_2{\mathrm{O}}\quad \left( {{\mathrm{RWGS}}} \right)$$

$$\Delta _{\mathrm{R}}{\mathrm{H}}_{{\mathrm{573K}}} = 38\;{\mathrm{kJ/mol;}}$$

R7 $${\mathrm{nCO}} + \left( {2{\mathrm{n}} + 1} \right){\mathrm{H}}_2 \to {\mathrm{C}}_n{\mathrm{H}}_{{\mathrm{2n + 2}}} + {\mathrm{nH}}_{\mathrm{2}}{\mathrm{O}}\quad \left( {{\mathrm{FTS}}} \right)$$

$$\Delta _{\mathrm{R}}{\mathrm{H}}_{{\mathrm{573K}}} = - 166\,{\mathrm{kJ/mol}}$$

(The values of Δ_R_H are reproduced from the literature^73,74^).

Prior to FTS, the RWGS reaction converts CO_2_ to CO to form dissociatively adsorbed *CO precursor molecules on the catalyst surface. Therefore, an understanding of the reaction conditions and catalytic performance for the RWGS reaction is necessary. Since RWGS (R6) is endothermic, it requires high temperatures for reasonable conversions. CO selectivity up to 100% can be achieved at 200–600 °C using the primary RWGS catalysts, such as Cu- and noble metal (Pt, Pd, Rh)-based catalysts, with CO_2_ conversions up to 50%^75^.

### Light olefins

Light olefins, including C_2_–C_4_ alkenes, are important target products for the FTS-based CO_2_ hydrogenation process. Fe-based catalyst systems are considered to be most suitable for olefin production by applying appropriate promoters, textural additives, or supports to form the required active sites for the different reaction stages. Fe and Co-based catalysts with alkali metal promoters (e.g., 35Fe–7Zr–1Ce–K^76^, Fe–Co/K–Al_2_O_3_^77^, and C–Fe–Zn/K^78^) are reported to be highly active for FTS to produce C_2+_ products^79,80^ with as high as 57% selectivity at 40–60% CO_2_ conversion (Fig. 2b). The most effective promoters are alkali metals, especially Na and K, as they limit the formation of methane while improving the selectivity to C_2+_ products^81^. Meiri et al.^82^ pointed out that the introduction of potassium can stabilize the texture of the Fe–Al–O spinel, increase the surface content of Fe_5_C_2_, and strengthen CO_2_ adsorption. In one study of fourteen different kinds of promoters added individually to MOF-derived Fe/C catalysts (Fig. 2e), only K dramatically enhanced the olefin selectivity from 0.7 to 36%^83^. Martinelli et al.^84^ concluded that the K-loading did not influence the CO_2_ conversion, but increased the olefin/paraffin ratio and the average molecular weight of the products. Besides the alkali metals, other metals such as Cu, Zn, Ni, Zr, Mn, and Pt can also be used to modify Fe-based catalysts^83^. For example, bimetallic Fe–Cu/Al_2_O_3_ catalysts can suppress CH_4_ formation and thus exhibit higher amounts of C_2_–C_7_ production compared with pure Fe/Al_2_O_3_ catalysts^85^.

Support material is another important factor that can influence catalyst activity and product selectivity by affecting the dispersion of active metals and the interactions between reactive intermediates and the support material. The support materials for Fe-based FT catalysts can be divided into metal oxides (e.g., ZrO_2_, CeO_2_, and Al_2_O_3_) and carbonaceous materials (e.g., MOFs, mesoporous carbon, carbon nanotubes (CNTs), graphene, and organic precursors to mesoporous carbon). Wang et al.^86^ studied several supports and found that a ZrO_2_ supported (K-Fe/ZrO_2_) catalyst exhibited the highest selectivity to lower olefins, while SiO_2_ was not suitable for the CO_2_-FTS reaction. Since Fe carbides are generally considered to be the active phase in FTS catalysis, carbon materials are naturally considered as good support materials for Fe catalysts; indeed, they have demonstrated excellent catalytic performance for olefin synthesis. Carbon support materials can improve the dispersion of the active metals and lead to higher selectivity to olefins^87^. An Fe-based core–shell nanocatalyst with Fe_3_O_4_ and Fe_5_C_2_ in the core and partially graphitized carbon in the shell was prepared to efficiently convert CO_2_ to C_2_–C_4_^=^ olefins. The olefin/paraffin ratio was increased by carefully controlling the content of carbon to improve the accessibility of reactants to the active sites^88^. CNTs and graphene are also good carbon supports with superior thermal and chemical stability^87,89^. For example, a honeycomb-structured graphene (HSG) with a special meso-macroporous architecture was devised to confine K-promoted Fe NPs for the CO_2_-to olefins reaction. The FeK1.5/HSG catalyst can increase the selectivity of C_2_–C_4_^=^ to 59.0% and the stability to 120 h, due to K promotion and HSG confinement effects^89^. Similarly, Ding et al. have successfully improved the catalytic activities by combining the K promoter and ZrO_2_ support with sufficient oxygen defects^90^.

### C_5+_ products

Liquid fuels (e.g., gasoline and diesel) and other value-added chemicals (e.g., aromatics and isoparaffins) are also desired products from CO_2_ hydrogenation via FTS. Jiang et al.^91^ prepared Fe- and FeCo-based catalysts and achieved, when promoted with an appropriate amount of K, ≥54.6% CO_2_ conversions and ≥47.0% C_5+_ selectivity (Fig. 2c). A Na–Fe_3_O_4_/HZSM-5 catalyst with three types of active sites was developed by Wei et al., demonstrating that 22% of CO_2_ can be directly converted to gasoline with a selectivity of 78%^17^. When HZSM-5 was changed to HMCM-22, aromatization was suppressed, while isomerization was promoted due to the appropriate Brønsted acid properties of HMCM-22 and the special lamellar structure consisting of two independent pore systems. Thus, 57% selectivity of isoparaffins was obtained over the Na–Fe_3_O_4_/HMCM-22 catalyst, while the values over Na–Fe_3_O_4_/HZSM-5 and Na–Fe_3_O_4_ were only 34 and 6%, respectively^92^. Similarly, aromatics are important feedstocks with applications in the synthesis of various polymers, petrochemicals, and medicines. ZnFeO_*x*_–*n*Na/HZSM-5 catalysts can achieve 75.6% selectivity to total aromatics at a CO_2_ conversion of 41.2% (Fig. 2f)^93^.

Owing to superior ability for chain growth, stability, and lower activity for the WGS reaction, Co-based catalysts have also been applied to produce long-chain C_5+_ hydrocarbons. Recently, a pure Co-based catalyst without promoters was reported to display high performance for both CO-FTS and CO_2_-FTS^94,95^. The Co/MIL-53(Al) catalyst was first found to exhibit 47.1% CO conversion and 68.6% selectivity to C_5+_ products. When it was applied to CO_2_-FTS, 35.0% selectivity to C_5+_ was still achieved at 260 °C. Furthermore, different kinds of promoters and support materials are employed to tune the product distribution towards heavier hydrocarbons. Shi et al. prepared a promoted Co–Cu bimetallic catalyst for CO_2_ hydrogenation based on consideration of Cu as a popular RWGS catalyst. For example, with the introduction of Na as a promoter to a CoCu/TiO_2_ catalyst, the CH_4_ selectivity significantly decreased from 89.5 to 26.1%, while the C_5+_ selectivity increased from 4.9 to 42.1%, with an excellent stability of more than 200 h^96^. He et al.^97^ reported the bimetallic Co_6_/MnO_x_ nanocatalyst with synergism between Co and Mn for 15.3% CO_2_ conversion and 53.2% C_5+_ selectivity.

### Higher alcohols

Reports of higher alcohol (C_2+_OH) production are less prevalent compared to methanol and other C_2+_ products of CO_2_ hydrogenation. However, C_2+_OH alcohols have higher energy density and wider applications as fuel additives. The formation of higher alcohols is a parallel reaction competing with formation of hydrocarbons during the FTS process, resulting in much lower selectivity to C_2+_OH. Furthermore, the synthesis of C_2+_OH is difficult, as both C–C coupling and OH formation are required. Interestingly, water was found to enhance the selectivity of higher alcohols to 82.5% over a 1 wt% Pt/Co_3_O_4_ catalyst (Table 1, Entry 34)^98^. Moreover, the C_2+_OH selectivity was affected by the volume fraction of water, increasing dramatically with small amounts (<20 vol%) of added water, and then decreased slightly with further addition of water (Fig. 2d). The promoting effect of water was attributed to assisting methanol dissociation to form more *CH_3_ species. *CH_3_ then can react with *CO to form *CH_3_CO, which will be further hydrogenated to ethanol (EtOH). Also, ordered Pd–Cu NPs, with charge transfer between Pd and Cu, are reported to efficiently convert CO_2_ to ethanol with 92.0% selectivity (Table 1, Entry 35). The rate-determining step of *CO hydrogenation to *HCO can be promoted on a Pd–Cu NPs/P25 catalyst, leading to lower *CO coverage and weaker *CO adsorption. Similarly, Cu–Fe and Zn–Fe interactions in the Fe-doped K/Cu–Zn catalyst have synergistic interactions, which can facilitate catalyst reduction and metal dispersion, as well as increase the yield of higher alcohols^99^. The reduction temperature can be regulated to obtain different phase compositions over the Co-based catalysts. For example, an alumina-supported catalyst reduced at 600 °C (CoAlO_*x*_−600) contained coexisting Co–CoO phases and exhibited 92.1% selectivity to ethanol at 140 °C, due to the formation of acetate intermediates from formate by insertion of *CH_*x*_^100^.

### Reaction mechanisms

Although many successful catalysts have been developed for the CO_2_-FTS reaction, the nature of the active sites and reaction mechanism remain controversial. In view of the catalysts reported to date, the mechanism of the RWGS reaction can be assigned to either the decomposition of *HOCO intermediates or to direct C–O bond cleavage to produce *CO^75^. Ko et al.^101^ pointed out that CO_2_ can be activated to anionic CO_2_^δ−^ through charge transfer from the pure and bimetallic alloy surfaces with bending of the structure and splitting of the π orbital. Both the adsorption energy of CO_2_^δ−^ and the reaction energy for CO_2_ dissociation are a linear function of adsorption energies of *CO and *O. To facilitate further *CO hydrogenation, the interaction between *CO and the catalyst interface need to be enhanced; otherwise, *CO is desorbed with concomitantly increased CO selectivity. According to the most-accepted reaction mechanism, during the FTS process, steps including *CO dissociative chemisorption on the active sites with hydrogen-assisted insertion to form *CH_*x*_ intermediates, initiation of chain growth through the coupling of *CH_*x*_, and the termination of chain growth through further hydrogenation, dehydrogenation, or insertion of non-dissociatively adsorbed *CO happen successively on the catalyst surface (Fig. 2a). *CO intermediates can be produced through the RWGS reaction, followed by the FTS reaction. According to modeling and kinetic analysis by Willauer et al., the RWGS reaction rate can reach 3.5 × 10^5^ s^−1^ initially and decreases to 0.032 s^−1^ within 2 s, while the FTS reaction rate is zero initially and increases to 0.004 s^−1^ at 18.7 s^102^. The FTS reaction is the rate-limiting step due to the much lower reaction rate. The FTS reaction during the CO_2_ hydrogenation process is limited by the reaction between the adsorbed CO and H_2_ to form *HCO intermediates, as reported by Pour et al.^103^ after evaluating different kinetic models and applying a genetic algorithm (GA) approach and the Levenberg–Marquardt (LM) method. Thus, efficient active sites for the FTS reaction are important for the whole CO_2_ hydrogenation process. Also, chain propagation in FTS is still in dispute based on the basic C–C coupling mechanisms^104^. The most limiting process or factor during FTS-based CO_2_ hydrogenation that should be addressed in future research may be affected significantly because of the various thermodynamic and physical properties of the reactions. For example, when using higher reaction temperatures (>300 °C), mass transfer appears to become the rate-limiting step instead of the surface reaction at relatively low temperatures^105^.

Concerning the active sites in iron and cobalt catalysts, several phases are present and change dynamically during reaction. The catalyst transformation in structure and composition has several steps with their own kinetic regimes^106^. The reduced iron catalysts mainly consist of α-Fe and Fe_3_O_4_ with extremely low activity at the beginning of FTS. Fe_3_O_4_ is capable of activating CO_2_ to CO via the RWGS reaction^81,107^. Then, the α-Fe reacts with the dissociatively adsorbed CO to form iron carbide (e.g., Fe_5_C_2_), generating Fischer–Tropsch activity. Thus, the catalytic sites of iron carbides appear to be active for CO activation and chain growth^81^. Considering the different functions of iron species and the zeolites’ superior properties of acid sites for oligomerization/aromatization/isomerization, a multifunctional structure Na–Fe_3_O_4_/HZSM-5 catalyst was developed and achieved as high as 78% selectivity to gasoline, with only 4% methane generated^17^. Therefore, researchers need to focus not only on optimizing the composition and structure of the catalysts, but also on developing new catalytic systems with desired properties by using comprehensive approaches that can directly control the microstructures of the catalysts and be used for more accurately identifying the reaction pathways during FTS-based CO_2_ hydrogenation.

**Fig. 3:**
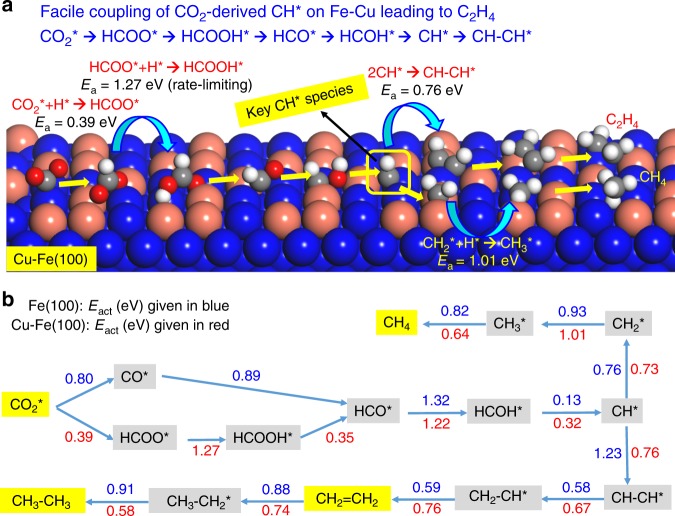
Mechanistic insight into C–C coupling over Fe-Cu bimetallic catalysts. **a** Mechanism of CO_2_ hydrogenation to C_2_H_4_ over a Cu–Fe(100) surface. **b** Reaction pathways for production of CH_4_, C_2_H_4_, and C_2_H_6_ from CO_2_ hydrogenation on Fe(100) and Cu–Fe(100) surfaces at 4/9 monolayer coverage. The kinetic barrier for each elementary step is given in eV. (Reprinted with permission from Nie et al. ^108^. Copyright (2017) American Chemical Society).

It is generally accepted that bulk Fe catalysts show insufficient catalytic performance and that the addition of promoters can enhance the selectivity to C_2+_ products. However, the essential basis of the promotion effect remains unclear. More investigation needs to focus on the reaction mechanism. For example, the reaction pathways over Fe/Al_2_O_3_, Cu/Al_2_O_3_, and Fe–Cu/Al_2_O_3_ catalysts are different, resulting in different product distributions^85^. Cu has high activity for the RWGS reaction instead of the CO_2_ methanation reaction, leading to more surface *CO. Thus, bimetallic Fe–Cu/Al_2_O_3_ catalysts form more CO, which leads to more C_2+_ hydrocarbons compared to the Fe/Al_2_O_3_ catalyst. Nie et al. also investigated the mechanism of CO_2_ hydrogenation to methane and C_2_ hydrocarbons over Fe(100) and Cu–Fe(100) surfaces and showed that the hydrogenation barrier of CH_2_* species is higher than those for C–C coupling and CH–CH* conversion to ethylene on Cu–Fe bimetallic catalysts (see Fig. 3)^108^, which results in a more selective process to ethylene. The addition of K to Fe–Cu/Al_2_O_3_ catalysts can further inhibit methanation and enhance the production of olefin-rich C_2_–C_4_ hydrocarbons by increasing the surface coverage of carbon^85^. DFT calculations show that the presence of K on Fe-based surfaces can enhance the CO_2_ adsorption strength and reduce the CO_2_ dissociation barrier (e.g., 2.36 eV for oct2-Fe_3_O_4_(111) versus 1.13 eV for K/oct2-Fe_3_O_4_(111))^109^. Therefore, the introduction of promoters can modify the surface electronic features.

## Outlook

### Methanol reaction based CO_2_ hydrogenation

The primary challenge in converting CO_2_ to value-added products is low selectivity due to excessive formation of CO and the large amount of water (H_2_O) generated. CO and CH_4_ are both formed due to partial reduction and cracking, respectively, during dehydration at the elevated temperatures (≥400 °C) generally employed for dehydration. To date, it has limited conversion (<30%) towards desired products (Table 1, Entries 1–5, 7–13). In addition, H_2_O produced from CH_3_OH synthesis and dehydrative coupling further complicates separations and, in some cases, deactivates the catalyst. However, altering the zeolite structure has been shown to alter product distributions and increase selectivity to desired products, which was realized by increasing the space velocity during catalytic CO_2_ hydrogenation over In_2_O_3_ combined with zeolites^26,110^. Through the introduction of CO conversion promoters, such as In–FeK, In–CoNa, or CuZnFe/zeolites, bifunctional catalysts can be upgraded to reduce CO formation. In addition, these catalytic CO_2_ conversion technologies face several other challenges, such as revealing the relationship between product type and molecular sieve type, increasing the CO_2_ conversion and final product yield via improving the structures of bifunctional catalysts, as well as further elucidating the reaction mechanisms. More detailed information on these points is depicted below.

First, more research on the zeolite properties is needed. By varying the zeolite structure, the product distribution can be better controlled. For example, HZSM-5 zeolite is selective towards DME and gasoline, whereas SAPO molecular sieves are preferred for light olefin generation. However, the structure-property relations between zeolite properties and CO_2_ hydrogenation activity have not yet been systematically investigated. Because many variables and conditions exist during zeolites synthesis (e.g., synthesis template, crystallization temperature, and Si/Al ratio), the relationship between zeolite properties and CO_2_ hydrogenation activity remains an active area of study^111–113^. Shape-selective catalysis was identified as a factor in these catalysts, as SAPO zeolite windows allowed only small linear hydrocarbons to pass, while H-ZSM zeolite windows allowed much larger branched and linear hydrocarbons to leave^114^. In addition, DFT calculations have linked density of surface Lewis acids and defects in the zeolite structure with dehydration activity^115,116^.

**Fig. 4:**
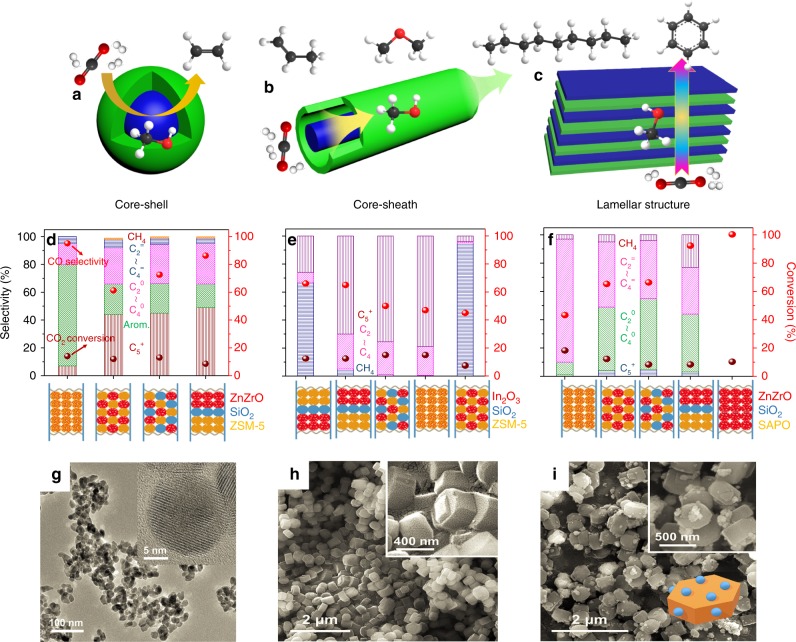
The structure and performance of bifunctional catalysts. **a**–**c** Proposed structures of bifunctional catalysts. **d**–**f** Hydrogenation of CO_2_ over bifunctional catalysts, which are integrated methanol synthesis catalyst and zeolites, with different spatial arrangements. (Adapted with permission from Li et al.^71^. Copyright (2019) Elsevier); Gao et al.^26^. Copyright (2017) Springer Nature; Li et al.^16^. Copyright (2017) American Chemical Society). **g**–**i** TEM and SEM images of bifunctional catalysts. (Reprinted with permission from Li et al.^71^. Copyright (2019) Elsevier).

Second, additional research on the structure of bifunctional catalysts is required. To improve CO_2_ conversion and final product yield, the morphology and structure of the bifunctional catalysts should be optimized, rather than just making physical mixtures. The precise control of the desired morphology and structure of the catalyst is a significant and synthetically challenging task (see Fig. 4a–c), as shown by the example of utilizing core–shell structures incorporating multiple-metal oxide cores to direct the growth of zeolite shells. Additionally, catalysts with core–sheath and lamellar structures are reported to exhibit high activity and stability for C=O bond hydrogenation^117^. Bifunctional catalysts can be assembled via layer-by-layer growth methods and probably exhibit better performance. Furthermore, efforts to improve important intrinsic factors, like the dispersion and crystallite size of active metals on methanol synthesis catalysts and the acid strength together with acid sites on the zeolites, as well as the methods of integration of the active components, should be made. As shown in Fig. 4d–f, different product distributions were obtained when the methanol synthesis catalyst and the zeolite were synthesized with different spatial arrangements^16,26,71^. The TEM and SEM images in Fig. 4g–i indicate that ZnZrO particles can be highly dispersed on the surface of HZSM-5 and maintain their own structures^16,26,71^. Sufficient physical mixing of the two components seems to be better than the granular form or a dual-bed separated in space by a layer of quartz sand.

Analyzing the integration between methanol synthesis catalysts and zeolites remains a challenge. DFT calculations may be the most efficient method to explore the interactions between CH_3_OH synthesis catalysts and dehydration catalysts. However, the information gained from DFT calculations can be limited because the systems studied are under idealized conditions, which are different from the ones in a packed bed reactor. As computing power with machine learning continues to increase, more complex and realistic catalytic systems can be modeled, which provides deep insight into the mechanism of CO_2_ hydrogenation to olefins, such as the design of 3D nanocrystal tandem catalysts with multiple interfaces for CO_2_ hydrogenation.

### FTS-based CO_2_ hydrogenation

The major obstacles of FTS-based CO_2_ hydrogenation lie in the thermal stability of CO_2_ and the complicated reaction mechanism with a wide product distribution. With the RWGS process being endothermic and FTS being exothermic, both need to be efficiently catalyzed under the same conditions, which sets a very strict requirement for the catalytic system. The designed catalysts should be effective for the RWGS reaction and also active enough for the subsequent FTS reaction. Different active sites must be precisely tuned and carefully dispersed on the support materials. These challenges will be discussed in view of different kinds of products: olefins, C_5+_ hydrocarbons, and higher alcohols.

The first, challenge to be discussed is production of light olefins. In spite of the promising results discussed, major challenges still remain. For example, the selectivity of C_2_–C_4_^=^ can be above 80% for catalysts with methanol as the reactive intermediate, while only about 50% selectivity to C_2_–C_4_^=^ is achieved on the catalysts for CO_2_ conversion via FTS (Table 1, Entries 14–21). The key to improve the C_2+_ selectivity is to develop catalysts that have compatible bifunctional active sites for the two reaction steps, namely, *CO generation and subsequent *CO hydrogenation. The appropriately engineered reaction window is extremely important. The consequence of the incompatible activity of these two different types of active sites could be high CH_4_ selectivity (~30%, Table 1, Entries 14–21), as is suggested by the activation energy of RWGS being higher than that of CH_4_ formation (81.0 and 59.3 kJ/mol, respectively) for Fe-based catalytic CO_2_ hydrogenation^105^. As shown in Table 1 (Entries 1–8, 14–23), the reaction temperature (~320 °C) for the CO_2_-FTS process is lower than that for the methanol-mediated route (~380 °C). Thus, the CO selectivity (~30%) is dramatically reduced due to relatively low temperatures for the RWGS reaction, leading to improved hydrocarbon selectivity.

Many researchers have tried to provide possible solutions for improving C_2+_ selectivity by adjusting the structures and compositions of the catalysts. Specifically, development of enhanced promoters, supports, and experimental conditions have been attempted. For example, bulk Fe is favorable for methane formation; however, olefin and long-chain hydrocarbon production can be enhanced with the addition of promoters (i.e., alkaline promoters). Potassium, as an electronic promoter, can regulate the phase proportion of Fe^0^/FeO_*x*_/FeC_*x*_ to maintain an optimum balance. The dissociative adsorption of *CO can be improved, while surface *H is decreased by donated electrons to the vacant d-orbital of Fe. The resulting higher C/H ratio favors chain growth and chain termination by forming unsaturated hydrocarbons^118^. Thus, a very important challenge is to increase the surface C/H ratio. Competitive adsorption between H_2_ and CO occurs on the catalyst surface. The partial pressure of CO is always limited due the difficulty of converting CO_2_ via the RWGS reaction. Since a high C/H ratio is desired to form unsaturated hydrocarbons, a possible strategy would be to enhance CO_2_ activation and CO adsorption, while weakening H_2_ adsorption^101^. In addition, the H_2_ ratio in the feed gas should also be carefully chosen. To achieve good results from FTS-based CO_2_ hydrogenation, more consideration is needed based on the characteristics of those reactions instead of imitation of the CO hydrogenation process.

To increase selectivity to light olefins, further hydrogenation must be suppressed. Thus, synergy between the promoter and the support is suggested. For example, an Al_2_O_3_ support can interact with a K promoter to form a KAlO_2_ phase under calcination temperatures above 500 °C, which has been shown to suppress further hydrogenation of olefins^77^. To increase the synergistic effect, the active metal can be mixed with supports. A series of K-modified supports (activated carbon, TiO_2_, ZrO_2_, SBA-15, B-ZSM-5, and Al_2_O_3_) were physically mixed with Fe_5_C_2_ and tested for direct hydrogenation of CO_2_. The Fe_5_C_2_–10 K/α-Al_2_O_3_ catalyst converted 40.9% of CO_2_ with a selectivity of 73.5% to C_2+_ products, containing 37.3% C_2_–C_4_^=^ and 31.1% C_5+_^119^. A promising support for Fe-based catalysts is MOF-derived carbon materials. Some researchers have recently developed methods of using MOFs to fabricate carbon supported Fe-based catalysts^83,120^. Two Fe-MOF-derived catalysts have been reported for CO_2_ hydrogenation, with high stability and selectivity to light olefins and liquid fuels^120,121^. However, the challenge of separating light olefins from unreacted CO_2_ and H_2_ remains. Increasingly advanced techniques and materials are required for their separation.

The second, set of challenges are production of C_5+_ products. These challenges are similar to those for the synthesis of light olefins, due to the shared fundamental reaction mechanism. The selectivity of C_5+_ products is also comparable to that of light olefins synthesis (Table 1, Entries 22–31). CO_2_ hydrogenation to C_5+_ products consist of multiple steps, including the RWGS reaction, C–C coupling, and acid-catalyzed reactions (oligomerization, isomerization, or aromatization). Therefore, cooperation between steps is required to develop an efficient and multifunctional catalyst. As zeolites are often used to incorporate the active sites, they generally undergo deactivation as a result of coke deposition. For example, the selectivity of isoparaffins over a Na–Fe_3_O_4_/HMCM-22 catalyst was reduced from ~60 to ~30% and the total acidity of HMCM-22 decreased from 0.200 to 0.032 mmol_pyridine_ g_cat_^−1^ after 12 h of reaction time, which was attributed to coke formation and accumulation in the cavities and channels^92^. Thus, a big challenge for the synthesis of C_5+_ products is to reduce coke deposition in the zeolites and to enhance the catalyst stability. As heavier hydrocarbons contain many components, it is difficult to precisely control a specific type of product. Zeolites with different framework topologies are suggested to regulate product distribution. In addition, in contrast to the CO-based FTS process, the reaction is fed with stable CO_2_ molecules; therefore, the reformation of CO_2_ from the produced CO via the WGS reaction is a thermodynamically favorable process, which limits CO_2_ conversion^17,122^. One method to decrease the reformation of CO_2_ from CO is to develop better multifunctional catalysts by employing appropriate promoters to facilitate the formation of iron carbide, which is known as the active catalyst for heavy hydrocarbon formation in FTS^96^, and to enhance the chemisorption and dissociation of CO_2_^123^. Also, by cycling reactants, CO_2_ conversion is increased. Therefore, finding suitable multifunctional catalyst combinations or promoters and optimizing the reactors should be an attractive area of research in the future. Dual-promoter systems are usually designed to improve the RWGS reaction activity, while increasing FTS activity and C_5+_ selectivity. Finally, there is a significant gap between the quality of synthesized liquid fuels and commercial gasoline. The octane number would be enhanced by developing catalysts that increase the fraction of isoparaffins in the gasoline.

Finally, the challenges to produce higher alcohols are considered. For higher alcohols synthesis, the major obstacles are the formation and insertion of the hydroxyl group during the C–C coupling process in the presence of parallel reactions. This is made even more challenging since the formation pathways are different over Fe- and Co-based catalysts and the mechanisms are controversial. On K/Fe/N-functionalized CNT catalysts, alcohol synthesis was explained by the reaction between hydrocarbon species, such as alkylidenes (R-CH_2_-CH = ), and *OH, which forms from the dissociation of adsorbed CO (*CO) and H_2_ (*H)^73^. *CO was hypothesized to react with *CH_3_ species to form *CH_3_CO, which was then hydrogenated to form CH_3_CH_2_OH on a Na-Co/SiO_2_ catalyst. To increase the yield of higher alcohols, efficient Co-based catalysts with more stable cobalt carbide (Co-Co_2_C) interfaces and/or Co-M alloy nanocrystals should be developed. Co_2_C is responsible for CO adsorption on the surface, whereas the Co metal is useful for CO dissociation and subsequent carbon-chain growth^124^. The synthesis of higher alcohols requires precise coordination between C–C coupling and OH formation, otherwise, more methanol or long-chain hydrocarbons would be produced. Also, the synergy of high metal dispersion and a high-density of hydroxyl groups on the supports can promote the selectivity to ethanol because the hydroxyls are able to stabilize formate species and protonate methanol^18^. The work from Fan’s group also indicates that metal oxyhydroxides, such as FeOOH^125^, TiO(OH)_2_^1^, and ZrO(OH)_2_^126^ can accelerate the CO_2_ sorption and desorption processes, thus reducing the energy required for CO_2_ capture based on experimental and DFT calculations. These metal oxyhydroxides are likely catalyst candidates because they contain hydroxyl groups that should enhance CO_2_ hydrogenation to higher alcohols.

### Transformational technologies for catalyst development

**Fig. 5:**
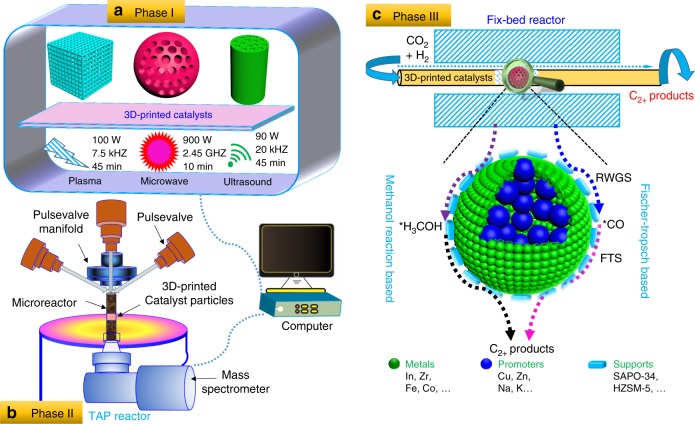
Scheme of AI-guided development of CO_2_ hydrogenation catalysts. Phase I is to prepare and modify catalysts using 3D printing technologies and new material modification technologies, such as plasma, microwave, and ultrasound modification^135,137,138^. Phase II is to use advanced techniques to characterize the catalysts. Phase III is to perform AI-guided evaluation of the catalysts.

Revolutionary material manufacturing, characterization, and evaluation technologies emerging in the new century may be instrumental for hydrogenating CO_2_ to high-value products. However, these technologies are only beginning to be explored for their application to CO_2_ hydrogenation. Thus, this perspective endeavors to fill in critical blanks. The keys are to discover alternative catalysts, modify current catalysts for CO_2_ activation, develop methods to prepare specialized catalysts on a large-scale, and intelligently evaluate catalysts. Guided by reaction theories and the associated heterogeneous catalytic CO_2_ hydrogenation experimental results, the feasibility of transformational technologies in three phases of CO_2_ hydrogenation will be explored: catalyst preparation and modification, characterization, and AI-guided evaluation (see Fig. 5).

Phase I is CO_2_ hydrogenation catalyst preparation and modification. Conventional labor-intensive lab-based CO_2_ hydrogenation catalyst preparation may be replaced by low-cost 3D-printing approaches to include characteristics of high mechanical strength and surface-to-volume ratio, with precise control of porosity, size, and shape^127^. The development of 3D-printed catalysts with designable and tunable structures is appealing and potentially useful for catalyst synthesis on a large scale. Although green 3D-printing has been utilized in preparing highly active, reusable, and stable catalysts for CO_2_ removal, methane combustion, methanol to olefins conversion, syngas methanation, and N-aryl compound synthesis^128–131^, it has not been used for CO_2_ hydrogenation or CO_2_ conversion catalysts to date. Also, most of the printed catalysts are millimeter- to micrometer-sized materials, while nano- or atomic-sized catalysts are still challenging to print by existing 3D printing technology. As the functional components of active CO_2_ hydrogenation catalysts are further studied (e.g., metals, promoters, and supports), more effort can be devoted to incorporating them into a fully integrated platform with diverse microstructures by 3D printing. As different spatial arrangements in bifunctional catalysts influence catalytic performance, 3D printing can assemble the components with multiple structures in their preferred configuration. Therefore, 3D printing provides an alternative approach for preparation of novel CO_2_ hydrogenation catalysts, especially for synthesizing bifunctional catalysts. Realizing that AI has proven to be a great tool in a range of fields, including materials discovery and design^132^, the authors intend to apply a pioneering outlook with its application to CO_2_ hydrogenation catalyst preparation, especially in combination with 3D printing technology. The 3D printing strategy already has advantages in potential scale-up manufacturing of catalysts, and AI technology would accelerate its application. AI-assisted 3D printing technologies can open new possibilities for the large-scale conversion of CO_2_.

Also, new material modification technologies, such as microwave, ultrasound, and plasma treatments, have been suggested to modify catalysts for CO_2_ activation, especially if the particle sizes or metal dispersion are affected by the 3D printing technologies. The utilization of unconventional modification tools in catalyst preparation and treatment can improve active phase dispersion to obtain a more uniform morphology with smaller particle sizes compared to the untreated catalysts^133–135^. Smaller particle size would enhance specific surface area and the exposed active sites available. The catalyst mechanical strength and the average basic site strength for CO_2_ adsorption can be increased by microwave irradiation treatment, due to its better heat transfer^133^. Catalysts synthesized with the assistance of ultrasound, microwave, or plasma treatments have been employed in CO_2_ hydrogenation and CO_2_/CH_4_ reforming reactions^134,136^. These unconventional methods need high frequency and input power with reduced processing time^135,137,138^. In addition, the reactor can be constructed with plasma or microwave enhancement. The CO_2_-to-CH_3_OH reaction was accomplished in a plasma reactor without heating or adding pressure, generating a CH_3_OH selectivity of 53.7% and a CO_2_ conversion of 21.2% over a Cu/γ-Al_2_O_3_ catalyst^136^. Coupling of plasma and catalyst lowered the kinetic barrier and energy cost associated with conventional high temperature and high pressure processing. Therefore, the use of these new modification technologies before or after 3D printing can possibly remedy defects introduced by 3D printing technologies.

Phase II is to characterize the CO_2_ hydrogenation catalysts. New material characterization technologies, including in situ scanning transmission electron microscopy and temporal analysis of product reactors, coupled with qualitative and/or quantitative species identification, such as gas chromatography–mass spectrometry, are suggested for characterization of the catalysts prepared with state-of-the-art and transformational technologies. To date, in situ CO_2_-temperature programmed surface reaction and diffuse reflectance infrared Fourier transform spectroscopy have been applied widely to elucidate how CO_2_ molecules dynamically interact with the catalyst. However, other characterization methods for heterogeneous catalysts under in situ conditions are challenging due to the lack of experimental facilities. Therefore, in the future, AI-assisted CO_2_ hydrogenation catalyst characterizations are needed. Machine learning especially can bring advanced computational techniques to the forefront of characterization of heterogeneous catalysts. For example, machine learning can be used to help interpret experimental spectra. Although the imaging and spectroscopic results are complicated, it would be helpful to design new catalysts and link models and experiments by connecting these results to structure–function information^139^. Timoshenko et al.^140^ have successfully deciphered the 3D geometric structure of supported Pt NPs with the use of X-ray absorption near-edge structure (XANES) spectroscopy and supervised machine learning. They solved the structures and reconstructed the morphology of Pt NPs just from the experimental XANES data using an artificial neural network. Therefore, theoretical simulations can assist in obtaining more spectra and deconvoluting the structural characterization.

Phase III is to perform AI-guided evaluation of CO_2_ hydrogenation catalysts. The prospect of using AI for identification of reaction intermediates and pathways and establishing kinetic models would be promising. First, the AI-guided method is expected to predict catalytic performance and to discover promising catalyst candidates. AI-based catalyst evaluation might largely help predict and improve catalyst stability, which however will require more significant effort. Zahrt et al.^141^ pointed out that machine learning has the potential to change the way chemists select and optimize catalysts. Some examples of recent results in AI-based catalyst evaluation follow. The hydrocarbon selectivities for DME conversion can be accurately predicted by an AI model with an *R*^2^ greater than 0.98 compared to experimental results^142^. Several known and unknown electrocatalysts for CO_2_ reduction and hydrogen evolution were identified from 1499 intermetallics by machine learning^27^. Specifically, 258 candidate surfaces among 102 alloys have been identified from 23,141 adsorption sites for the H_2_ evolution reaction.

Currently, it is inconvenient to characterize catalyst structures in situ during stability tests. Thus, in situ monitoring of the catalyst structure dynamics and transformation by AI would be beneficial. Second, we need more automatic, integrated, and flexible set-ups to evaluate catalysts. Third, data analysis could be performed and kinetic models evaluated with the help of AI. Numerous experimental data for CO_2_ hydrogenation have been generated and this existing data should be reanalyzed. Kitchin suggests that machine learning can be applied to build models with more sophisticated methods^143^, generating new relevant data and calculated properties. Furthermore, it is crucial to identify reaction intermediates and pathways by AI. A simple syngas reaction on Rh(111) exhibits more than 2000 potential pathways^144^, in comparison with the more complicated CO_2_ hydrogenation reaction routes that are strongly influenced by the various active centers. Hence, it is necessary to analyze the reaction mechanism by employing machine learning and DFT calculations.

Finally, predicting relationships among the characteristics of the catalysts, such as Lewis acidity, CO_2_ conversion efficiency, and product selectivity, with AI based on the available experimental data and DFT computation results will be helpful for discussing structure–function relationships and accelerating the discovery of catalytic mechanisms. Although AI offers many promising applications in CO_2_ hydrogenation, robust and versatile AI is still in a primitive stage. A difficulty for AI development comes from the slowly developing applications of big data and a convenient language or software. Li et al.^28^ pointed out that it is crucial and challenging to build additional criteria for screening and synthesizing catalysts, to quantify the uncertainties of machine-learning models, and to develop fingerprints of more sophisticated active sites with targeted functionalities. Also, more data for machine learning are required, including all reactions related to CO_2_, such as FTS, CO_2_/CH_4_ reforming, and CO_2_ hydrogenation. In conclusion, the collaborative efforts from experts and scientists in catalysis and materials design, machine-learning practitioners, and algorithm developers all over the world are needed to promote its development.
